# Arsenic Levels in Chicken: Nachman et al. Respond

**DOI:** 10.1289/ehp.1307083R

**Published:** 2013-09-01

**Authors:** Keeve E. Nachman, Patrick A. Baron, Georg Raber, Kevin A. Francesconi, David C. Love

**Affiliations:** 1 Johns Hopkins Center for a Livable Future; 2 Department of Environmental Health Sciences, and; 3 Department of Health Policy and Management, Bloomberg School of Public Health, Johns Hopkins University, Baltimore, Maryland, USA; 4 Institute of Chemistry, Karl-Franzens University, Graz, Austria, E-mail: knachman@jhsph.edu

In our paper (Nachman et al. 2013) we focused on dietary arsenic exposure from the use of arsenic-based drugs in food animal production, specifically chicken. We thank Lasky for broadening the discussion on arsenic regulations for food to include federal agencies beyond the Food and Drug Administration (FDA).

The results of our study (Nachman et al. 2013) indicate that the use of arsenic-based drugs increases the levels of inorganic arsenic in chicken meat. Based on these findings, we recommend banning the use of arsenic-based drugs in food animal production, which are under the jurisdiction of the FDA. It is important, however, to recognize the potential role that could be played by the U.S. Department of Agriculture (USDA) Food Safety and Inspection Service (FSIS) under its National Residue Program (NRP).

Under its mandate, the NRP facilitates the monitoring of arsenic levels in poultry products and supports enforcement actions for animal products in violation of arsenic standards (USDA FSIS 2012). Unfortunately, the NRP faces constraints (in addition to those noted by Lasky) that limit its effectiveness (Silbergeld and Nachman 2008). The most important of these constraints is the current arsenic standard for meat, which was set before 1963 (FDA 1963) and does not account for recent epidemiologic research. In addition, the standard applies to total arsenic concentrations rather than to inorganic arsenic, the species of greatest health relevance. Because arsenic can be present in food in various forms that have widely varying toxicity, standards might need to be species specific.

The U.S. Environmental Protection Agency (EPA) is currently revising its toxicological assessment for inorganic arsenic (U.S. EPA 2010) as part of its Integrated Risk Information System (IRIS) program. The purpose of this revision is to produce health-based guidance that can be useful in setting arsenic standards in different media (including foods) that reflect our current understanding of dose–response relationships between arsenic exposures and adverse health outcomes. To achieve this goal, coordination between the FDA, U.S. EPA, and NRP is essential. By applying appropriate standards and methods with adequate sensitivity for the arsenic species of interest, the NRP could play a central role in minimizing dietary exposure to arsenic through animal products.

Although sale of roxarsone remains suspended in the United States, nitarsone, a chemically similar arsenical drug, continues to be sold (Zoetis 2013). Industry statements in the media have confirmed nitarsone use in the turkey industry (Aubrey 2013), and the USDA estimates of per capita turkey consumption are increasing (USDA 2013). Research is needed to characterize potential contributions of nitarsone to inorganic arsenic concentrations in turkey meat. For these reasons, monitoring efforts remain relevant. In the absence of regulations that limit inorganic arsenic in our foods, the banning of arsenic-based drugs would minimize dietary arsenic exposures in poultry consumers.
